# How to Investigate the Effect of Music on Breathing during Exercise: Methodology and Tools

**DOI:** 10.3390/s22062351

**Published:** 2022-03-18

**Authors:** Lorenzo Innocenti, Andrea Nicolò, Carlo Massaroni, Carlo Minganti, Emiliano Schena, Massimo Sacchetti

**Affiliations:** 1 Department of Movement, Human and Health Sciences, University of Rome “Foro Italico”, Piazza Lauro de Bosis 6, 00135 Rome, Italy; l.innocenti@studenti.uniroma4.it (L.I.); carlo.minganti@uniroma4.it (C.M.); massimo.sacchetti@uniroma4.it (M.S.); 2 Unit of Measurements and Biomedical Instrumentation, Department of Engineering, Università Campus Bio-Medico di Roma, Via Alvaro del Portillo, 21, 00128 Rome, Italy; c.massaroni@unicampus.it (C.M.); e.schena@unicampus.it (E.S.)

**Keywords:** respiratory sensors, mechanical sensors, acoustic sensors, entrainment, respiratory frequency, respiratory rate, music tempo, music features, respiratory signal, cycling

## Abstract

Music is an invaluable tool to improve affective valence during exercise, with the potential contribution of a mechanism called rhythmic entrainment. However, several methodological limitations impair our current understanding of the effect of music on relevant psychophysiological responses to exercise, including breathing variables. This study presents conceptual, methodological, and operational insight favoring the investigation of the effect of music on breathing during exercise. Three tools were developed for the quantification of the presence, degree, and magnitude of music-locomotor, locomotor-breathing, and music-breathing entrainment. The occurrence of entrainment was assessed during 30 min of moderate cycling exercise performed either when listening to music or not, and was complemented by the recording of relevant psychophysiological and mechanical variables. Respiratory frequency and expiratory time were among the physiological variables that were affected to a greater extent by music during exercise, and a significant (*p* < 0.05) music-breathing entrainment was found in all 12 participants. These findings suggest the importance of evaluating the effect of music on breathing responses to exercise, with potential implications for exercise prescription and adherence, and for the development of wearable devices simultaneously measuring music, locomotor, and breathing signals.

## 1. Introduction

Music pervades many aspects of sport and exercise [1]. Some forms of physical activity are inextricably linked with music (e.g., dance, ice skating, rhythmic gymnastics, and synchronized swimming) while many others may benefit from it in terms of exercise pleasure and even performance improvement [1]. This explains the ever-growing research interest in investigating the effects of music on exercising humans. A recent meta-analysis found a moderate and positive effect of music on affective valence, and a small but significant effect on perceived exertion and oxygen uptake (VO_2_) while no beneficial effects of music were generally found for heart rate (HR) [1]. However, music has the potential to substantially affect other physiological variables that have been less considered in the context of exercise, such as breathing variables, including respiratory frequency (*f*_R_) and its subcomponents.

Investigating the effects of music on breathing during exercise may prove to be of great value for two main reasons. First, *f*_R_ is a good marker of physical effort and exercise tolerance in different conditions and populations [2,3,4,5,6]. Second, *f*_R_ is, to a great extent, regulated by non-metabolic inputs that may be modulated by music, including emotions and arousal (see the next section). The physiological rationale for an effect of music on *f*_R_ during exercise is further supported by findings from experimental studies evaluating this relationship both at rest [7,8,9,10,11,12,13] and during exercise [14,15,16]. Some of these studies have suggested that *f*_R_ increases with music tempo [7,8,9,12,14,16], but a series of methodological issues raise concerns about this interpretation. For instance, Bernardi et al. [7] increased the music tempo by selecting music tracks with different genres, thus inevitably modulating other music features as well. The same issue applies to exercise studies, where the effects of slow or fast music tempo were assessed by proposing music tracks with different characteristics [14,16]. These and other methodological problems make our understanding of the effect of music on breathing during exercise limited.

Several researchers have suggested that the effect of music on *f*_R_ may be mediated by an entrainment between the music tempo and the breathing rhythm, although this issue has been predominantly addressed at rest [7,8,9,10,17]. The fact that music may entrain breathing is supported by neurophysiological evidence, and the so-called rhythmic entrainment appears to mediate the positive effect of music on affective valence [18], with potential implications for exercise adherence. However, until rhythmic entrainment is objectively quantified during exercise, any improvement in our understanding of this issue would be prevented. Hence, in this study, we have proposed an advanced version of a tool that we had preliminarily developed for evaluating the occurrence of music-breathing entrainment during exercise [19], and have presented it alongside the music-locomotor and locomotor-breathing entrainment tools, for a more comprehensive assessment of the rhythmic entrainment phenomenon. To favor the use of these tools, we have also developed a conceptual and an operational framework describing how music can affect *f*_R_ and how the underlying mechanisms can be investigated. Furthermore, we have provided an example of how to design an experimental study aiming at assessing the effect of music on breathing during exercise, thus clarifying how relevant findings should be interpreted to improve our understanding of this issue.

### 1.1. Conceptual Framework Describing the Mechanisms Underlying the Effect of Music on Breathing during Exercise

**Figure 1:**
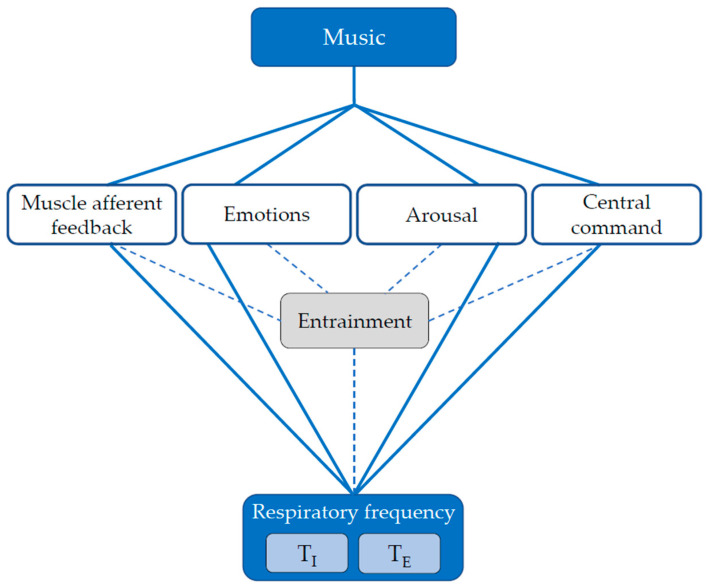
Conceptual framework describing the mechanisms underlying the effect of music on respiratory frequency, inspiratory time, and expiratory time during exercise. Note that the four listed inputs may regulate *f*_R_ and its subcomponents (T_I_ and T_E_) either via entrainment (dashed lines) or not (solid lines).

Evidence suggests that the two components of minute ventilation (V_E_), i.e., *f*_R_ and tidal volume (V_T_), are to some extent regulated by different inputs during exercise [2,5,20,21,22,23]. Indeed, *f*_R_ and V_T_ have been defined as the behavioral and metabolic components of V_E_, respectively [5,23]. The differential control of *f*_R_ and V_T_ has important implications for the investigation of the effect of music on breathing, as music can to a large extent be regarded as a behavioral intervention. The notion that music has the potential to affect *f*_R_ rather than V_T_ is also supported by experimental findings [15,16]. This explains why we primarily deal with the mechanisms regulating *f*_R_ during exercise, with a special focus on the inputs that can be modulated by music, i.e., muscle afferent feedback, emotions, arousal, and central command (see Figure 1). It has even been suggested that inspiratory time (T_I_) and expiratory time (T_E_) may to some extent be regulated by different inputs [24,25], hence supporting their separate evaluation. We therefore provide a brief description of how each of these inputs may be modulated by music during exercise.

It is well recognized that music modulates emotions and affective states [18,26,27,28], thus potentially affecting *f*_R_. Indeed, the fact that emotions influence *f*_R_ is well established [5,29,30]. Furthermore, music affects the state of arousal [28], which is also a regulator of *f*_R_. This proposition is supported by the description of a “wakefulness drive to breathe” involving an increase in central neural activity or arousal, which preferentially affects *f*_R_ rather than V_T_ [31,32,33]. It is also well documented that increases in arousal induced by cognitive tasks preferentially affect *f*_R_ [34]. The known influence of music on areas related to motor control [18] suggests that music may also affect *f*_R_ by manipulating central command (the activity of motor and premotor areas of the brain relating to volitional/motor control), which is a major regulator of *f*_R_, at least during high-intensity exercise [2,20,22,23]. A music-mediated regulation of central command is also indirectly supported by data suggesting that perceived exertion is affected by music [1], with perceived exertion mainly being regulated by central command [35,36]. Music may also affect the motor drive to the locomotor muscles—and the muscle afferent feedback—by favoring a change in the locomotor rhythm [37,38]. Variations in the locomotor rhythm may affect *f*_R_ both via modulations of central command and muscle afferent feedback, although the latter may provide a greater relative contribution to *f*_R_ at low to moderate intensities [39]. Any change in the locomotor rhythm may also affect the metabolic rate [39,40]. Hence, we anticipate that music may have an indirect ‘metabolic’ effect on physiological responses, although its influence on *f*_R_ is not expected to be substantial, with *f*_R_ being largely driven by non-metabolic inputs [2,5,20,21,22,23].

Importantly, each of the four inputs may affect *f*_R_ entraining the breathing rhythm or not. Entrainment can be defined as the process through which two physical or biological systems become synchronized by virtue of interacting with each other [18]. In the context of music, entrainment involves neural coupling at different cortical and subcortical levels, involving motor, cognitive, perceptual, and emotional processes [18,28,41]. While these concepts may appear elusive, fine measurements of music, breathing, and locomotor signals provide us with the opportunity to unravel some of the mechanisms underlying the effect of music on breathing during exercise, including the assessment of various forms of entrainment. This constitutes the physiological premise for the development and use of the entrainment tools presented in this study.

### 1.2. Tools for Investigating the Effect of Music on Breathing during Exercise

**Figure 2:**
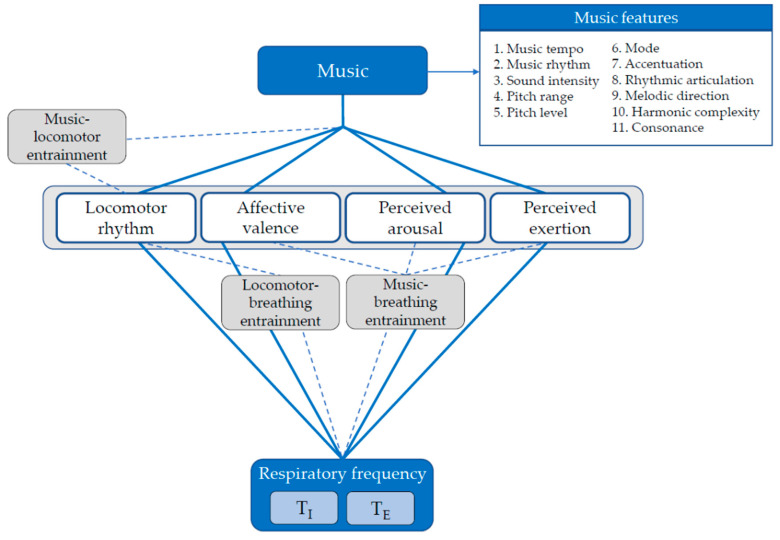
Operational framework providing information on the tools to use to investigate the mechanisms underlying the effect of music on *f*_R_ and its subcomponents. The grey rectangular box highlights how the tools reported inside should be evaluated together for providing information on the magnitude of the inputs regulating *f*_R_. For instance, the combined evaluation of affective valence and perceived arousal provides insight into the emotions experienced during exercise. The three entrainment tools proposed in this study and their potential connections are depicted. Note that each music track can be described by the means of 11 features. The features from 1 to 5 can be quantified objectively and can therefore be easily controlled when selecting music for experimental purposes. The features from 6 to 11 are more influenced by subjective perception of music. See Gomez and Danuser [11] for detailed information on the music features.

Previous studies on the effect of music on psychophysiological responses to exercise present several methodological issues that we have attempted to address by providing conceptual and operational insight. A music track can be described by different features that should be carefully considered when designing a study or prescribing music in practical exercise settings. However, this issue has often been overlooked. We have anticipated how several studies have suggested that music tempo may affect *f*_R_ both at rest and during exercise [7,9,12,14,16], but none of them have investigated the effect of music tempo per se because other music features have not been controlled adequately. Hence, we emphasize that the selection of music should carefully take into account the 11 music features proposed by Gomez and Danuser [11] and reported in Figure 2. The authors have shown how the manipulation of the music features differently affects breathing and affective valence at rest [11] while similar findings during exercise have not been provided so far.

Along with a careful selection of music tracks based on their music features, researchers are advised to measure useful variables that may provide insight into the mechanisms regulating *f*_R_ and its subcomponents. We have anticipated above how pedaling cadence may provide some information on the contribution of muscle afferent feedback to *f*_R_ regulation, especially during moderate exercise. However, other inputs may underly the effect of cadence on *f*_R_ [39], which suggests caution when interpreting the effect of music on pedaling cadence and physiological variables. Further information can be retrieved using valid scales measuring sensations associated with emotions, arousal, or central command. The Feeling Scale is largely used to assess affective valence (also during exercise), and provides information on the pleasure or displeasure perceived during a given task [42]. The Felt Arousal Scale measures perceived arousal [43], and may thus be used as a proxy of the magnitude of arousal or activation. The combined use of the Feeling scale and the Felt Arousal Scale may further help in the description of the emotions experienced during exercise [28,44]. Besides, perceived exertion has long been used as a marker of central command [35,36]. The Borg 6–20 rating of perceived exertion (RPE) scale is one of the scales that can be used to monitor RPE [45]. In the discussion section, we provide an example of how data acquired with these tools can be interpreted from a mechanistic perspective.

Given the long-standing proposition that music may entrain physiological responses [10,18,46], we have developed some tools evaluating whether entrainment occurs and to what extent. We propose the use of three different entrainment tools that collectively allow us to understand whether music entrains the locomotor rhythm, the breathing rhythm, or facilitates locomotor-breathing coupling. While the following sections provide a detailed description of how to use these three tools, here we briefly anticipate their importance from conceptual and practical perspectives. The music-locomotor entrainment tool assesses whether an imposed music tempo entrains the locomotor rhythm, thus allowing for an objective quantification of a concept described in the literature as “synchronization” [47], which has so far not been addressed at the level of detail proposed in this study. Indeed, while Karageorghis and colleagues [26,27] merely account for the ratio between the music tempo and the locomotor rhythm, our approach evaluates whether the effect of music influences the development of each pedaling revolution. The music-locomotor entrainment tool may also help assess how music tempo affects locomotor-breathing entrainment, which is a phenomenon that has been investigated extensively in the field of exercise physiology [39,48]. The locomotor-breathing entrainment tool evaluates whether single breaths are entrained by the locomotor rhythm, and some studies suggest that acoustic cues may facilitate locomotor-breathing coupling [46,49]. The music-breathing entrainment tool assesses whether an imposed music tempo influences the development of each breathing cycle, thus providing quantitative insight into an otherwise elusive link between the areas of the brain influenced by music and those regulating breathing. Collectively, the three entrainment tools shed some light on the so-called rhythmic entrainment [18,28,41], with important implications for exercise enjoyment and adherence. In the following sections, we present a study designed to provide an example of how to use these tools.

## 2. Materials and Methods

### 2.1. Participants

In total, 12 male participants (mean ± SD: age 23 ± 3 years, height 180.6 ± 4.5 cm, body mass 78.8 ± 9.7 kg) volunteered to participate in this study. They were recreationally active (not specifically trained in cycling) with no specific music skills. All participants gave written informed consent according to the Declaration of Helsinki. The experimental protocols were approved by the Institutional Review Board of the University of Rome “Foro Italico” (CAR 90/2021). Participants were asked to abstain from vigorous exercise and alcohol and caffeine consumption for at least 24 h before each laboratory visit.

### 2.2. Experimental Overview

All tests were completed in a quiet laboratory with a room temperature of 19–21 °C and at the same time of the day (±2 h). Participants presented to the laboratory on 2 separate occasions over a period of 1 or 2 weeks, with visits separated by at least 48 h. They performed 30 min of moderate cycling exercise either when listening to music or not. In the music condition, 6 music tracks of 5 min were played over a period of 30 min. Cycling exercise was performed on an electromagnetically braked cycle ergometer (Lode Excalibur Sport, Groningen, The Netherlands) that was adjusted individually according to the anthropometric characteristics of the participants. Physiological, psychological, and mechanical variables were measured during cycling as described below.

### 2.3. Experimental Protocol

**Figure 3:**
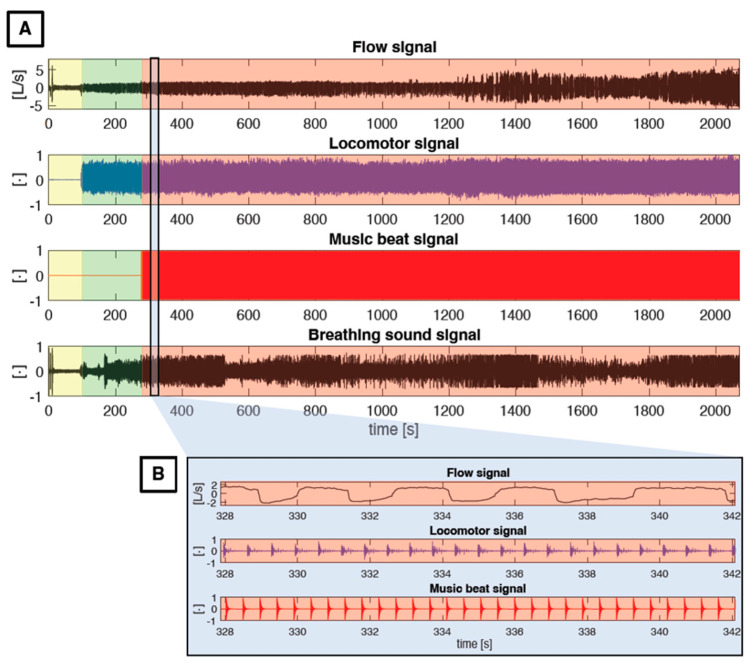
Schematic representation of the different phases of the experimental protocol and the main signals recorded (**A**). The music beat signal allows for the identification of the start of music listening. (**B**) shows an enlarged portion of the three signals used for the analyses of entrainment.

The experimental protocol lasted 35 min and is depicted in Figure 3. During the first 2 min, participants were asked to perform 3 deep breaths immediately followed by 5 s of apnea to synchronize data acquired with the Ableton 10 Suite music software (Berlin, Germany) with those recorded with the metabolic cart (Quark CPET, Cosmed, Rome, Italy), as previously detailed [19]. Subsequently, participants performed 3 min of baseline pedaling at 20 W followed by 30 min of constant work rate cycling exercise, where the power output was individualized based on body mass according to the formula: power output (W) = 1.2·body mass (kg). No restrictions on pedaling cadence were imposed and no feedback or encouragement were provided to participants. Cardiorespiratory and mechanical variables were measured continuously throughout the experimental protocol while psychological variables were measured every 5 min during the 30 min bout of moderate exercise.

### 2.4. Music Selection and Administration

Two music tracks were carefully selected by an experienced musician to provide differences in music rhythm while keeping other music features constant, such as music tempo (i.e., 128 bpm), sound intensity (i.e., 72.1 dB), pitch range (wide), pitch level (high), and mode (major). The music rhythm was outstanding for “Superwave” of Ummet Ozcan (electro-house music genre; hereinafter ‘House’) and excessively vague for “Oyo como va” of Carlos Santana (rock-Latin music genre; hereinafter ‘Latin’). Both music tracks received a motivational grade above 36 points from all the participants as assessed by the Brunel Music Rating Inventory-3 [47]. To differentiate the effect of the music rhythm from that of the music tempo on the ventilatory responses to exercise, the music tempo was altered for both House and Latin by reproducing each music also at 120 and 136 bpm. The combination of music genres (House and Latin) and levels of music tempo (120, 128, and 136 bpm) resulted in 6 different music tracks (i.e., House_120_, House_128_, House_136_, Latin_120_, Latin_128_, and Latin_136_) that were played during the 30 min of moderate exercise. The order of administration of the music tracks with different tempo was randomized across participants, but an alternation between House and Latin tracks was always maintained.

The music tracks were treated with careful editing to enhance the quality of the music sound delivered by the AirPods (Apple, California) using the following Ableton audio tools: the EQ Eight tool was used for equalization in the low frequencies (20–100 Hz) and high frequencies (5–10 kHz), the Multiband Dynamics tool was used to equalize the volume power in the most deficient frequency ranges, and the Spectrum tool was used to monitor the correct distribution of equalization. Both music proposals were uploaded on Ableton and played with a higher audio compressed resolution of 320 kbps.

### 2.5. Music Signals and Data Processing

**Figure 4:**
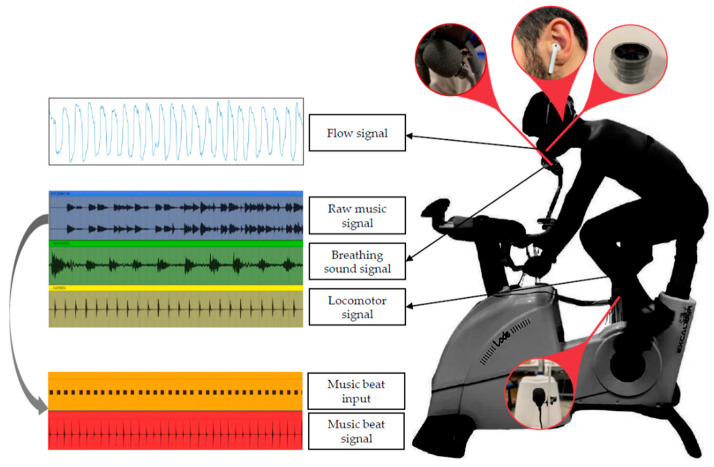
Graphical description of the sensors used in this study and the related signals. Note that, after data acquisition, the raw music signal was visually inspected by an experienced musician for the identification of each music beat (music beat input), and then converted into the music beat signal for further analysis.

As previously described [19], the raw music signal was recorded with the Ableton 10 Suite software (Berlin, Germany) and was visually inspected by an experienced musician that manually inserted the notes corresponding to each music beat (one-quarter of the tempo) in an additional music channel (music beat input). This process of manual input has proven to be very accurate, as previously shown experimentally [19]. The music beat input was then converted into a music beat signal for the automatic identification of the music beats needed to perform the entrainment analyses. Figure 4 provides a graphical representation of the conversion of the raw music signal into the music beat signal. The identification of music beat events was subsequently performed by identifying all the peaks in the music beat signal. After the normalization of the signal between 0 and 1, the analysis was carried out on the whole signal for each subject by using a fixed time and amplitude threshold (minimum peak between 2 consecutive events equal to 300 ms, minimum amplitude of 10%). A visual inspection was then carried out to verify the correct identification of all the peaks.

### 2.6. Respiratory Signals and Data Processing

As previously described [19], two breathing signals were simultaneously recorded, i.e., the flow signal and the breathing sound signal (see Figure 3 and Figure 4). The flow signal (in L/s) was collected with a metabolic cart (Quark CPET, Cosmed, Rome, Italy) at a sampling rate of 50 Hz and processed offline in the MATLAB^®^ environment to compute the locomotor-breathing and the music-breathing entrainment, as described below. In order to synchronize the flow signal with the music signal, the breathing sound signal was also recorded with a handheld condenser microphone (Gyvazla Microphone, Shenzheb, China) placed close to the distal orifice of the metabolic cart turbine, as shown in Figure 4. The breathing sound signal was acquired with Ableton 10 Suite (Berlin, Germany), thus guaranteeing synchronization with the music raw signal. It was exported at a sampling rate of 22,050 Hz. The above-described breathing manoeuvres (three deep breaths followed by apnea) were performed to synchronize the flow signal with the breathing sound signal, and consequently with the raw music signal. Thereafter, only the flow signal was considered for further analysis. The breathing volume was obtained by the approximate cumulative integral of the respiratory flow via the trapezoidal method with time-unit spacing. A band filter between 0.05 and 5 Hz was applied to remove breathing-unrelated events and noise and to emphasize the inspiratory and inspiratory events. This signal was then lowpass filtered with a moving average span equal to 20 samples, which allowed preserving of the trace even in case of respiratory rates above 80 breaths·min^−1^. All the maximum and minimum peaks were then identified on this signal to determine the end-inspiratory and end-expiratory events. The analysis was carried out on the whole signal—previously normalized between 0 and 1—for each subject using a fixed time and amplitude threshold (minimum peak between 2 consecutive events equal to 300 ms, minimum amplitude of 20%). In the case of multiple inspiratory (or expiratory) events between two expiratory (or inspiratory) events, the algorithm automatically removes the second or subsequent events (considered false positives) and considers only the first.

### 2.7. Cardiorespiratory and Ventilatory Variables

*f*_R_, T_I_, T_E_, V_E_, V_T_, VO_2_, carbon dioxide output (VCO_2_), respiratory exchange ratio (RER), and HR were measured breath-by-breath with a metabolic cart (Quark CPET, Cosmed, Rome, Italy) to describe the effect of music on ventilatory and other physiological variables. Breath-by-breath data were averaged over 5-min periods, which corresponds to the duration of each of the 6 music tracks.

### 2.8. Locomotor Signal and Data Processing

To measure pedaling cadence and identify the start of each pedaling cycle, a locomotor signal was recorded with a custom-made electromechanical system specifically devised to enable the synchronization of the locomotor signal with the music and breathing signals. Specifically, a handheld condenser microphone (Gyvazla Microphone, Shenzheb, China) was clipped to a mechanical support located at the top dead center of the cycle ergometer (see Figure 4). A guitar plectrum was attached on the upper part of the right pedaling crank with the purpose of determining an oscillation of the mechanical support at the start of each pedaling cycle. The oscillation of the mechanical support led to a rapid increase in the amplitude of the signal recorded with the microphone condenser, thus facilitating the identification of each pedaling cycle by analyzing the microphone signal. This is the typical response of a 2nd-order under-damped system. On this signal, after the normalization between 0 and 1, the maximum peak was automatically identified with a peak detector with a minimum threshold of 10% of the maximum amplitude and time threshold of 200 ms to automatically locate the start of each pedaling cycle.

### 2.9. Psychological Measures

Before data collection, participants were carefully familiarized with the Borg 6–20 RPE scale [45], the Felt Arousal Scale [43], and the Feeling Scale [42]. During the 30-min moderate exercise, the 3 scales were presented to participants every 5 min during the last 15 s of each music track.

### 2.10. Data Analysis

As a preliminary evaluation of the effect of music on physiological, psychological, and mechanical responses, values were averaged across participants irrespective of the specific music track played and were compared with the control condition at different time points (i.e., 5, 10, 15, 20, 25, and 30 min). To differentiate the effect of the music rhythm from that of the music tempo, values were also averaged across participants considering the specific music tracks (i.e., House_120_, House_128_, House_136_, Latin_120_, Latin_128_, and Latin_136_) instead of the time windows where they were played. Nevertheless, the time window was taken into account when comparing the specific music tracks with their respective control conditions. Given that randomization resulted in the 6 music tracks being played at different time windows across participants, each music track had a specific control condition accounting for the time where music was played for each participant (i.e., CON_House_120_, CON_House_128_, CON_House_136_, CON_Latin_120_, CON_Latin_128_, and CON_Latin_136_). This is why we first compared each music genre with its own control condition, and only later we directly compared House vs. Latin music tracks.

### 2.11. Entrainment

**Figure 5:**
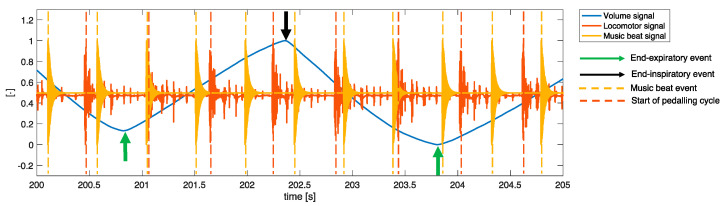
Graphical description of how the events used for the computation of entrainment were identified from the breathing volume signal, the locomotor signal, and the music beat signal.

Before performing any of the 3 entrainment analyses, the first and last 15 s of each of the 6 music tracks were removed to minimize any residual effect of the previous music and to exclude the time windows where participants were asked to rate affective valence, perceived arousal, and perceived exertion. The music-locomotor entrainment, the locomotor-breathing entrainment, and the music-breathing entrainment were calculated considering: (i) the 6 music tracks together (Total); (ii) the House and Latin music tracks separately; and (iii) the 3 levels of music tempo (i.e., 120, 128,and 136 bpm) separately. The main events detected to compute entrainment are graphically represented in Figure 5, and the details of the 3 analyses are provided below.

### 2.12. Music-Locomotor Entrainment

The music-locomotor entrainment tool presented here assesses the presence, magnitude, and degree of phase coupling between the music tempo and the pedaling cadence. It does not evaluate the ratio between the average values of music tempo and pedaling cadence, but it provides information on how each pedaling cycle develops as a result of an imposed music tempo. To this end, the music beat period (one quarter of tempo) was subdivided into 10 windows of equivalent durations to assess how the start of the pedaling cycle was distributed in each of them. In the absence of entrainment, it is expected that the number of pedaling cycles is evenly distributed in each of the 10 windows. Conversely, entrainment presents when the distribution of the start of the pedaling cycles is significantly different (*p* < 0.05) compared to an even distribution, as assessed by means of the chi-squared test. When a significant distribution was found, a *p* value and an effect size (phi) were computed for each of the 10 windows to identify those presenting high and significant percentages of occurrence of pedaling cycles. When significant values were observed in a single window, the % occurrence of pedaling cycles was used to describe the degree of entrainment and the corresponding phi was used to describe the magnitude of entrainment. When significant values were observed in two or more windows, we selected the highest phi to describe the magnitude of entrainment while the degree of entrainment was computed by summing the % occurrence of each window where significance was found. For instance, if 2 windows present 14.76% (phi = 0.39) and 14.29% (phi = 0.36) of occurrence, respectively, the degree of entrainment is 29.05% while the magnitude of entrainment can be represented by the highest phi observed (i.e., phi = 0.39). The effect size is small for phi > 0.10, medium for phi > 0.30, and large for phi > 0.50 [50]. Conceptually, the degree of entrainment provides information on the number of pedaling cycles that are entrained by music (expressed as a percentage) while the magnitude of entrainment is a measure of the effect of the music-locomotor entrainment, with phi being the effect size of the chi-squared test. Since the entrainment phenomenon may not occur in all participants, the average % degree (±SD) and the average phi (±SD) were computed considering only those reporting a significant entrainment. It is of note that both the *p* value and phi are influenced by the number of observations (number of pedaling cycles in this instance), which is crucial information to consider before designing studies or segmenting data to run the entrainment analysis.

### 2.13. Locomotor-Breathing Entrainment

The locomotor-breathing tool used in this study assesses the presence, magnitude, and degree of phase coupling between the locomotor and breathing rhythms. While it reproduces to a great extent the analysis introduced by Bernasconi and Kohl [48] and largely used in the field of exercise physiology, we amended this methodology to improve it from a statistical perspective. As described by Bernasconi and Kohl [48], we subdivided the pedaling cycle into 10 windows of equal durations and evaluated in which window the end-inspiration (or end-expiration) event falls for each breath cycle. Subsequently, we calculated the occurrence of the end-inspiration (or end-expiration) events across the 10 windows for all the breaths and compared it with an even distribution expected in the absence of entrainment, using the chi-squared test. The improvement in the methodology used by Bernasconi and Kohl [48] resides in the way we assessed statistical significance. The authors [48] suggested that entrainment is found when the occurrence of end-inspiration (or end-expiration) events is ≥15% in a given window. This 15% value was also used in other research studies as a cut-off value for considering the locomotor-breathing entrainment significant [51]. However, the *p* value of the chi-squared test is affected by the number of observations (number of breaths in this instance), and the use of a fixed cut-off value is therefore not advised. As an alternative solution, we used the chi-squared test, which provides a specific *p* value to be compared with alfa (*p* = 0.05). When significance was found, the *p* value and phi were computed for each of the 10 windows, and used as described above for the music-locomotor entrainment. A further advantage of the amendment proposed here is that the magnitude of entrainment can be assessed alongside the degree of entrainment, with the two parameters providing different and complementary information.

### 2.14. Music-Breathing Entrainment

The music-breathing tool used in the present study assesses the presence, magnitude, and degree of phase coupling between the music tempo and the breathing rhythm. In other words, the tool evaluates how the breathing cycle develops as a result of an imposed music tempo. We have previously presented a preliminary version of this tool [19] that has been substantially advanced in this study. The major changes proposed here relate to: (i) the identification of the music beat period; (ii) the statistical analysis used to determine entrainment; and (iii) the computation of entrainment for the end-expiratory events in addition to the end-inspiratory events. While Innocenti et al. [19] calculated music-breathing entrainment considering two-quarter of tempo as the music beat period, in this study, we assessed if one-quarter of tempo allows for an improved assessment of the music-breathing entrainment. Indeed, when the music beat period is subdivided into 10 windows with equal durations, each window of one-quarter tempo is half of the length of the windows of the two-quarter tempo, thus increasing the sensitivity of entrainment detection. This solution is further beneficial as it guarantees consistency with the music beat period used for the music-locomotor entrainment (i.e., one-quarter tempo). For both music beat periods, the entrainment analysis was performed in analogy with what we described above for the music-locomotor entrainment. The distribution of end-inspiratory (or end-expiratory) events across the 10 windows was compared with an even distribution by means of the chi-squared test. The level of significance, degree of entrainment, and magnitude of the effect were evaluated as reported for the other two entrainment tools.

### 2.15. Statistical Analysis

Statistical analysis was conducted using IBM SPSS Statistics 26 (SPSS Inc., Chicago, IL, USA). Data were checked for normality before analysis. A two-way repeated measures ANOVA was used to compare the time course of physiological, psychological, and mechanical variables between the music condition and the control condition. A two-way repeated measures ANOVA was also used to compare the House or Latin music tracks with their respective control conditions (i.e., CON_House or CON_Latin), separately. The same analysis was subsequently performed to directly compare the House and Latin music tracks. When the sphericity assumption was violated, the Greenhouse–Geisser adjustment was performed. Partial eta squared (*η*_p_^2^) effect sizes were calculated and interpreted as small for *η*_p_^2^ ≥ 0.01, medium for *η*_p_^2^ ≥ 0.059, or large for *η*_p_^2^ ≥ 0.138 [50]. When a significant interaction was found, the Student’s paired *t* test was used to compare the music and control conditions at each time point. A value of *p* < 0.05 was considered statistically significant in all analyses. The results are expressed as means (±SD) in the text and tables and as means (±SEM) in the Figures.

## 3. Results

### 3.1. Effects of Music on the Time Course of Physiological, Psychological, and Mechanical Variables

**Figure 6:**
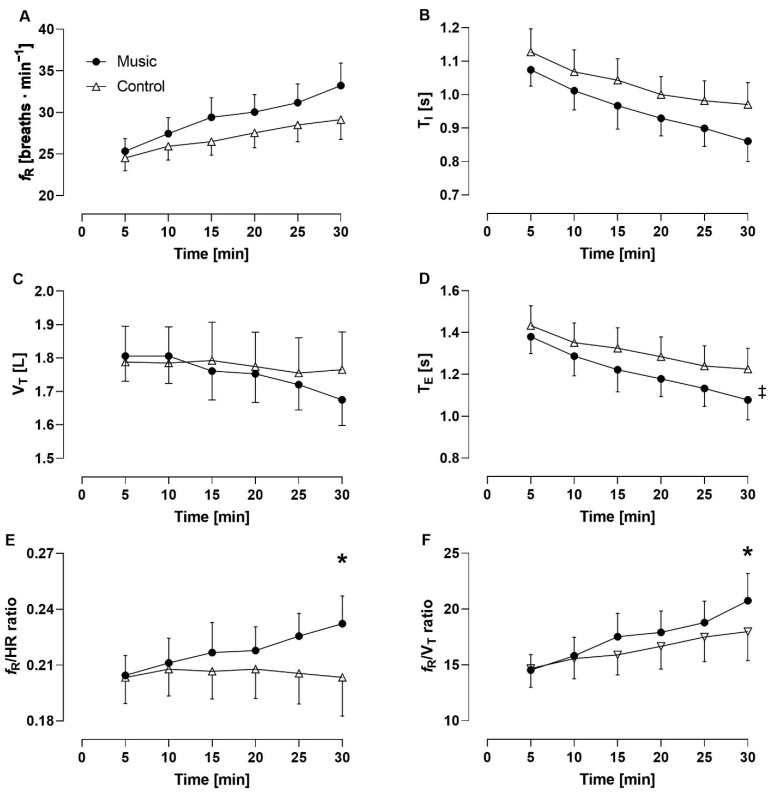
Time course of respiratory frequency (**A**), inspiratory time (**B**), tidal volume (**C**), expiratory time (**D**), *f*_R_/HR ratio (**E**), and *f*_R_/V_T_ ratio (**F**) for the music condition (filled circles) and the control condition (open triangles). ‡ main effect of condition (*p* < 0.05); * *p* < 0.05 vs. control condition.

**Figure 7:**
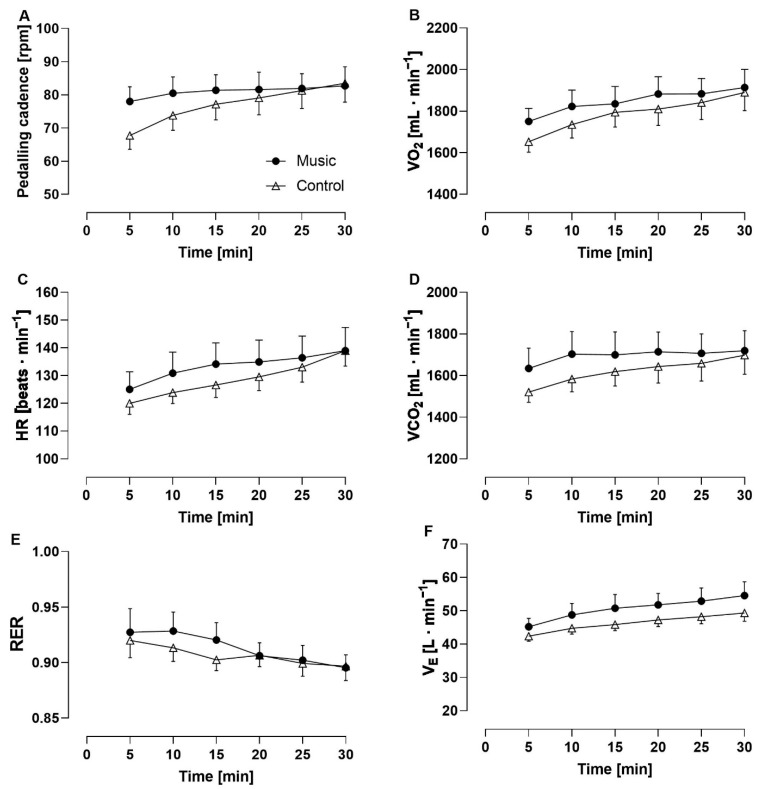
Time course of pedaling cadence (**A**), oxygen uptake (**B**), heart rate (**C**), carbon dioxide output (**D**), respiratory exchange ratio (RER) (**E**), and minute ventilation (**F**) for the music condition (filled circles) and the control condition (open triangles).

**Figure 8:**
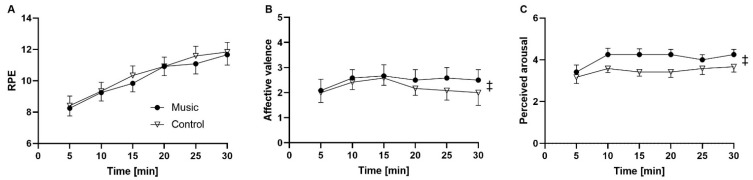
Time course of rating of perceived exertion (**A**), affective valence (**B**), and perceived arousal (**C**) for the music condition (filled circles) and the control condition (open triangles). ‡ main effect of condition (*p* < 0.05).

A significant condition × time interaction was observed for *f*_R_/V_T_ ratio (*p* = 0.008; *η*_p_^2^ = 0.243) and *f*_R_/HR ratio (*p* = 0.022; *η*_p_^2^ = 0.271). A statistical trend was observed for pedaling cadence (*p* = 0.58; *η*_p_^2^ = 0.243) while no significant interaction was found for the other variables. A main effect of condition was observed for T_E_ (*p* = 0.047; *η*_p_^2^ = 0.325), perceived arousal (*p* = 0.026; *η*_p_^2^ = 0.368), and affective valence (*p* = 0.028; *η*_p_^2^ = 0.375) but not for the other variables, except that *f*_R_ showed a statistical trend (*p* = 0.083; *η*_p_^2^ = 0.249). A main effect of time was observed for all the variables considered (*p* < 0.007; *η*_p_^2^ > 0.213), except for VCO_2_, which showed a statistical trend (*p* = 0.054; *η*_p_^2^ = 0.249), and V_T_, affective valence, perceived arousal, and *f*_R_/HR ratio, where no significant effect was found. Due to technical problems, HR data were analyzed for nine participants. The time course of physiological, psychological, and mechanical variables is provided in Figure 6, Figure 7 and Figure 8.

### 3.2. Effects of Music Rhythm vs. Music Tempo on Physiological, Psychological, and Mechanical Variables

**Figure 9:**
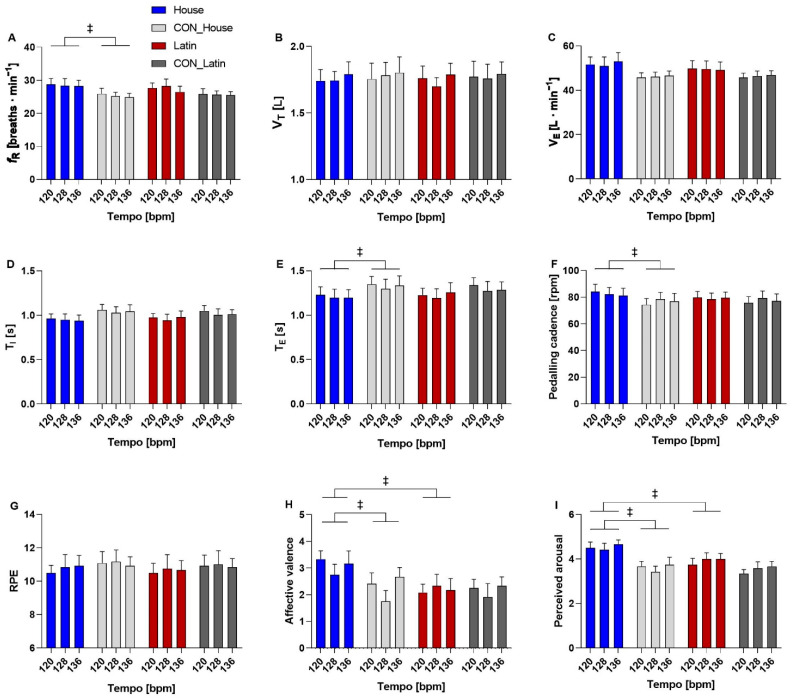
Comparison between House and control conditions (CON_House), Latin and control conditions (CON_Latin), and House and Latin conditions for respiratory frequency (**A**), tidal volume (**B**), minute ventilation (**C**), inspiratory time (**D**), expiratory time (**E**), pedaling cadence (**F**), rating of perceived exertion (**G**), affective valence (**H**), and perceived arousal (**I**). ‡ main effect of condition (*p* < 0.05).

When comparing the House music with its own control condition at the three levels of music tempo, no significant condition x music tempo interaction was found for any of the variables considered. Conversely, a significant main effect of condition was found for *f*_R_ (*p* = 0.037; *η*_p_^2^ = 0.338), T_E_ (*p* = 0.029; *η*_p_^2^ = 0.365), pedaling cadence (*p* = 0.027; *η*_p_^2^ = 0.372), affective valence (*p* = 0.001; *η*_p_^2^ = 0.620), and perceived arousal (*p* = 0.006; *η*_p_^2^ = 0.513). A statistical trend was observed for T_I_ (*p* = 0.079; *η*_p_^2^ = 0.254) and V_E_ (*p* = 0.061; *η*_p_^2^ = 0.285) while no significant effect was found for V_T_ and perceived exertion. These responses are depicted in Figure 9.

When comparing the Latin music with its own control condition at the three levels of music tempo, no significant condition × music tempo interaction nor main effect of condition were found for *f*_R_, T_I_, V_E_, V_T_, pedaling cadence, perceived exertion, affective valence, and perceived arousal while a statistical trend was found for the main effect of condition for T_E_ (*p* = 0.085; *η*_p_^2^ = 0.227). A comparison with data from previous studies is provided in Table 1.

When comparing the House music with the Latin music at the three levels of music tempo, no condition × music tempo interaction was found for any of the variables considered while a main effect of condition was observed for affective valence (*p* < 0.001; *η*_p_^2^ = 0.821) and perceived arousal (*p* = 0.015; *η*_p_^2^ = 0.429). A statistical trend was found for the main effect of condition for *f*_R_ (*p* = 0.075; *η*_p_^2^ = 0.260) and V_E_ (*p* = 0.085; *η*_p_^2^ = 0.245) while no effect was found for T_I_, T_E_, V_T_, pedaling cadence, and RPE.

### 3.3. Music-Locomotor Entrainment

A significant music-locomotor entrainment was found in 2 (Total, House, Latin 128 bpm and 136 bpm) or 3 (120 bpm) participants (Table 2 and Table 3), with an average degree of entrainment ranging from 14.1% (136 bpm) to 27.8% (120 bpm), and an average magnitude of entrainment ranging from 0.17 (Total) to 0.39 (120 bpm). Table 4 reports the number of pedaling cycles considered for the analyses.

### 3.4. Locomotor-Breathing Entrainment

When considering the 30-min moderate cycling bout, a significant locomotor-breathing entrainment was found in four participants both for end-inspiration and end-expiration events in the music condition while in the control condition, two participants showed a significant entrainment when considering end-inspiration events and one participant when considering end-expiration events. When the 30-min cycling protocol was partitioned according to music genre or music tempo, the number of participants with significant entrainment varied. More details are provided in Table 2 and Table 3.

### 3.5. Music-Breathing Entrainment

All the participants showed a significant music-breathing entrainment (see Table 5). Generally, a greater number of participants showed a significant music-breathing entrainment when considering one-quarter of music tempo compared to two-quarter of music tempo, and when considering end-expiratory events compared to end-inspiratory events. The highest number of participants with significant entrainment was found at 120 bpm (*n* = 7), where the average degree of entrainment was 18.5% and the average magnitude of entrainment was 0.47. Further details are provided in Table 2 and Table 3.

## 4. Discussion

The effect of music on psychophysiological responses to exercise can only be appreciated if its evaluation is performed considering a series of physiological, methodological, and technical aspects. To improve our understanding of the effect of music on breathing during exercise, we have presented a multidisciplinary approach providing conceptual and practical insight on how to address this issue. Specifically, we have: (i) provided a new conceptual framework identifying the mechanisms underlying the effect of music on ventilatory responses to exercise; (ii) presented new tools facilitating the assessment of the effect of music on breathing, including the music-locomotor and the music-breathing entrainment analyses; and (iii) provided an example of an experimental study aiming to investigate the effect of music on psychophysiological responses to exercise. Our findings show that *f*_R_ (and T_E_) is sensitive to the behavioral effect of music during exercise, with important implications for exercise prescription and adherence and for the development of wearable devices measuring *f*_R_ and its subcomponents in the context of exercise.

The evaluation of the effect of music on breathing during exercise has to consider the differential control of *f*_R_ and V_T_. We have previously suggested that *f*_R_ and V_T_ are the behavioral and metabolic components of V_E_, respectively [5,23], and this proposition is in line with the present findings. We observed an increase in *f*_R_ (and a decrease in T_E_) with music while no significant effect of music was found on V_T_, either when reporting its response over time or when comparing the House and Latin music tracks with their own control conditions. If music had any effect on V_T_, it was in the opposite direction of *f*_R_, as we found a significant interaction for the time course of the *f*_R_/V_T_ ratio but not for that of *f*_R_. These findings are in line with the notion that V_T_ is adjusted on the basis of *f*_R_ values in an attempt to meet metabolic requirements [22,23].

While we acknowledge the preliminary nature of the present findings, they are discussed here with the primary purpose of helping the reader understand how the tools presented in this study can be used to shed light on the mechanisms underlying the effect of music on breathing during exercise. An important advantage of the proposed methodology resides in the opportunity to partially unravel the complex effect of pedaling cadence on ventilatory responses. To this end, we developed an original tool that objectively evaluates whether the music tempo entrains the locomotor rhythm, thus providing new insight into the so-called “synchronization” phenomenon [47]. Indeed, while different studies have evaluated the presence or absence (i.e., asynchronization) of synchronization based on the ratio between the music tempo and the locomotor rhythm [47,52], our approach allows for the precise quantification of the presence (significant entrainment), degree, and magnitude of music-locomotor entrainment. The fine description of how the locomotor cycle develops as a result of an imposed music tempo is fundamental both from mechanistic and practical perspectives. From a mechanistic perspective, it provides information on how music affects locomotion and helps evaluate if this effect influences locomotor-breathing coupling. For instance, the small number of participants showing music-locomotor entrainment in this study suggests that the music-locomotor phenomenon did not contribute substantially to the differences in pedaling cadence observed between the music and control conditions. From a practical perspective, the opportunity of quantifying entrainment offers advantages in the prescription of music during exercise. Indeed, it has been suggested that entrainment may mediate the positive effect of music on affective valence [18,28,41], with implications for different populations (e.g., patients, young, and elderly) and purposes (exercise promotion, educational, leisure activity, and rehabilitation). Hence, the efficacy of the entrainment-based physical activities mentioned by Trost et al. [18] can be monitored objectively with the music-locomotor tool presented here.

The need to evaluate the music-locomotor entrainment together with the locomotor-breathing entrainment is reinforced by findings reporting a higher degree of entrainment when exercising with the acoustic cue of a metronome compared to a control condition [46,49]. However, these findings are not in line with our results because we found a relatively low occurrence of locomotor-breathing entrainment in both the music and control conditions. Hence, any effect of cadence on *f*_R_ can only to a minor extent be attributed to locomotor-breathing entrainment and even less to an effect of music-locomotor entrainment on the locomotor-breathing entrainment. It is also conceivable to expect no substantial ‘metabolic’ effects of music (via changes in pedaling cadence) on *f*_R_, unlike for V_E_, metabolic rate (VO_2_ and VCO_2_), and HR. Indeed, the average time course of these variables resembled the time course of pedaling cadence more than that of *f*_R_. Despite the different time courses of *f*_R_ and cadence, we observed a higher pedaling cadence in the House condition compared to its control condition, and this finding may contribute to explaining the higher *f*_R_. The effect of cadence on *f*_R_ may have been mediated by muscle afferent feedback more than central command because no effect of music on perceived exertion was observed in this study.

In the light of the conceptual and operational frameworks presented in the introduction, we can further explore the mechanisms underlying the effect of music on breathing by considering the role played by emotions and arousal. We consistently observed a higher affective valence and arousal in the conditions where *f*_R_ or T_E_ showed either significant values or a statistical trend. These findings are in line with the notion that the cortical and subcortical areas of the brain related with emotional processing, or influenced by the state of arousal, may affect *f*_R_ [29,31]. From this perspective, it is of note that we observed a significant music-breathing entrainment in all the participants, extending previous observations on the existence of this phenomenon at rest [10]. The occurrence of music-breathing entrainment allows us to advance the hypothesis that the behavioral effect of music on breathing may have partially been driven by the entrainment phenomena described by Trost et al. [18]. As such, the opportunity of objectively quantifying the degree and magnitude of the music-breathing entrainment opens interesting avenues from both neurophysiological and practical perspectives. For instance, it allows researchers to identify the music features maximizing the occurrence of music-breathing entrainment, if the methodological considerations provided above are taken into account. If entrainment plays an important role in mediating the positive effect of music on affective valence [18], it is fundamental to objectively measure entrainment in its various forms using the tools developed in this study.

Our findings appear not to support the proposition that *f*_R_ is largely affected by music tempo, as instead suggested by previous studies performed both at rest and during exercise [7,9,14,16]. However, these apparently contrasting findings should be evaluated in the light of some methodological considerations. None of the previous studies have attempted to evaluate if music tempo per se affects *f*_R_ while keeping the other music features unaltered. This issue can be addressed experimentally by using the same music track played at different music tempos, as we performed in this study. Conversely, the previous studies varied the music tempo by selecting music tracks with different music genres [7,9,14,16], hence providing evident changes in other music features as well. However, we acknowledge the relatively limited range of music tempos investigated in this study (from 120 to 136 bpm) that was not widened to avoid voice distortion. This issue could be solved by selecting a music track without lyrics (e.g., a movie soundtrack) and playing it at considerably different music tempos. We have also shown how the music rhythm can be manipulated while attempting not to substantially affect other music features, at least those that can be objectively quantified, such as music tempo, sound intensity, pitch range, and pitch level. While it is conceivable to suggest that the music-breathing entrainment may occur more frequently when listening to music tracks with a more outstanding rhythm [19], the preliminary nature of our findings do not allow us to speculate on potential differences in entrainment observed when listening to House compared to Latin music tracks. Further studies are needed to identify the music features that affect breathing variables and music-breathing entrainment, and researchers are encouraged to use the methodological framework provided in this study.

The present findings have important implications for the development of wearable systems facilitating the prescription of music during exercise and the evaluation of its effects. It is of note that *f*_R_ and T_E_ were among the physiological variables that were affected by music to a greater extent. This is further evident when evaluating the effect of music on the *f*_R_/HR ratio, which reveals how the behavioral effect of music influences *f*_R_ more than HR. Given the variety of contact-based sensors that can be used to monitor *f*_R_ [53] and the ever-growing diffusion of high-quality wearable solutions to reproduce music, our findings encourage the development of wearable devices enabling the synchronization of the music signal with the breathing and locomotor signals. The quality of the breathing signal should be sufficient for a valid measurement of the *f*_R_ subcomponents and for the identification of the end-inspiratory and end-expiratory events. The opportunity to provide valid measures of ventilatory variables and rhythmic entrainment with wearable devices has the potential to tailor the prescription of music to the needs of single individuals and to improve affective valence and exercise adherence, with potential health benefits for a variety of populations. The achievement of this goal requires the establishment of fruitful synergies between exercise physiologists, music experts, and bioengineers, as the multidisciplinary nature of this study highlights.

Given the importance of *f*_R_ as a marker of physical effort in different exercise protocols and conditions [2,3,4,5,6], the fact that music may not necessarily affect *f*_R_ and perceived exertion in the same way has implications for monitoring effort in fitness and sporting activities where music listening is common practice. While a partial dissociation between *f*_R_ and RPE is often observed during moderate exercise [22], the limited data available suggest that music may affect *f*_R_ more than RPE even at higher intensities (see Table 1). Further studies are required to evaluate whether *f*_R_ effectively reflects physical effort while listening to music in different exercise intensity domains and how exercise should be monitored when music is provided.

## 5. Conclusions

In this study, we have presented a multidisciplinary approach to improve our understanding of the effect of music on breathing during exercise. The complexity of music and its effects should not discourage researchers from investigating the mechanisms explaining how music can improve affective valence and exercise adherence or modulate psychophysiological responses to exercise. We therefore developed specific tools allowing for the objective quantification of the occurrence of different forms of rhythmic entrainment, which has been proposed as an important mediator of the positive effect of music on affective valence. The development of these tools allowed us to provide original data documenting the existence of the music-breathing entrainment phenomenon during exercise and to describe it in terms of presence, degree, and magnitude. We have also facilitated the use of these tools in future studies by providing new conceptual and operational frameworks explaining how music can affect breathing and how the mechanisms underlying this effect can be disentangled.

## Figures and Tables

**Table 1:** Comparison between the present study and previous studies investigating the effect of music on breathing during exercise.

Study	(*n*)	Exercise Conditions	Music Conditions	Music Tempo (bpm)	*f_R_* (breaths·min^−1^)	V_T_ (L)	V_E_ (L·min^−1^)	RPE
Birnbaum et al. [16]	11 (6 M, 5 F)	Constant-speed running	CON		40	1.6	63	13
			Slow music (4 songs)	97, 107, 83, 113	-	-	-	-
			Fast music (4 songs)	128, 123, 139, 106	↑	-	↑	-
Brownley et al. [14]	16 (4 M, 12 F)	Walking/running at low, moderate, and high intensities	CON		24; 30; 37	n.r.	n.r.	9; 11; 14
			Slow music (playlist)	n.r.	-; -; -	n.r.	-	-
			Fast music (1 of 2 playlists)	154–162	↑; ↑; ↑	n.r.	-	-
Maddigan et al. [15]	16 (8 M, 8 F)	High-intensity interval cycling	CON		39	2.1	82	16
			Music (playlist)	130	↑	-	-	-
The present study	12 M	Constant work rate moderate cycling	CON_House		27; 27; 27	1.8; 1.8; 1.8	46; 46; 46	11; 11; 11
			House	120; 128; 136	↑; ↑; ↑	-	-	-
			CON_Latin		27; 27; 27	1.8; 1.8; 1.8	46; 46; 47	11; 11; 11
			Latin	120; 128; 136	-	-	-	-

M, males; F, females; *n*, number of participants; CON, control condition (no music); *f*_R_, respiratory frequency; V_T_, tidal volume; V_E_, minute ventilation; RPE, rating of perceived exertion; average values of *f*_R_, V_T_, V_E_ and RPE in the control condition were reported when made available from previous studies (either in numeric or graphical format); n.r., not reported; -, not significantly different from CON; ↑, significant increase compared to CON. The three conditions in Brownley et al. [14] are low, moderate, and high intensity, respectively; those in the present study are 120, 128, and 136 bpm of music tempo, respectively.

**Table 2:** Presence, degree, and magnitude of entrainment for the music-locomotor, locomotor-breathing, and music-breathing entrainment analyses, when considering all the music tracks together or the House and Latin tracks separately.

Form of Entrainment	Music Beat Period/Condition	Total			House			Latin		
		Degree (%)	(*n*)	Phi	Degree (%)	(*n*)	Phi	Degree (%)	(*n*)	Phi
Music-locomotor	ONE	15.7 ± 5.8	2	0.17 ± 0.02	18.0 ± 9.0	2	0.18 ± 0.04	18.6 ± 7.6	2	0.24 ± 0.06
Locomotor-breathing	Music End_In	13.2 ± 0.9	4	0.28 ± 0.06	13.9 ± 0.2	3	0.33 ± 0.01	13.8 ± 0.7	3	0.32 ± 0.04
	Music End_Ex	15.7 ± 5.7	4	0.23 ± 0.09	13.8 ± 2.0	3	0.32 ± 0.14			
	CON End_In	18.0 ± 3.6	2	0.54 ± 0.14	25.5	1	0.25	14.5 ± 1.3	4	0.37 ± 0.08
	CON End_Ex	34.2	1	0.64	17.0 ± 6.3	6	0.37 ± 0.08	19.2 ± 8.2	5	0.29 ± 0.10
Music-breathing	ONE End_In	19.5 ± 9.0	2	0.39 ± 0.17	13.2	1	0.27	17.1 ± 5.8	5	0.42 ± 0.14
	ONE End_Ex	12.7 ± 0.5	6	0.24 ± 0.03	17.3 ± 6.4	4	0.40 ± 0.15	17.2 ± 5.1	4	0.39 ± 0.07
	TWO End_In	13.2	1	0.28	13.0	1	0.26			
	TWO End_Ex	12.4	1	0.22	13.4	1	0.30	18.4 ± 8.0	3	0.43 ± 0.19

ONE, one quarter of music tempo; TWO, two quarter of music tempo; End_In, end-inspiratory events; End_Ex, end-expiratory events; CON, control condition (no music); *n*, number of participants with significant entrainment (*p* < 0.05); blank spaces are provided when no significant entrainment was found for any of the participants.

**Table 3:** Presence, degree, and magnitude of entrainment for the music-locomotor, locomotor-breathing, and music-breathing entrainment analyses, when considering the three levels of music tempo separately.

Form of Entrainment	Music Beat Period/Condition	120 bpm			128 bpm			136 bpm		
		Degree (%)	(*n*)	Phi	Degree (%)	(*n*)	Phi	Degree (%)	(*n*)	Phi
Music-locomotor	ONE	27.8 ± 1.8	3	0.39 ± 0.11	26.6 ± 4.3	2	0.33 ± 0.24	14.1	1	0.35
Locomotor-breathing	Music End_In	14.9	1	0.40	14.5 ± 0.5	3	0.38 ± 0.04	14.3 ± 0.6	2	0.37 ± 0.03
	Music End_Ex	16.2	1	0.49	13.7 ± 0.7	3	0.31 ± 0.05	16.7	1	0.52
	CON End_In	22.5 ± 8.5	2	0.44 ± 0.08	20.3 ± 9.5	3	0.38 ± 0.04	19.7 ± 7.4	3	0.41 ± 0.06
	CON End_Ex	23.0 ± 9.1	5	0.52 ± 0.05	23.2 ± 6.4	2	0.49 ± 0.21	29.1	1	0.39
Music-breathing	ONE End_In	15.0 ± 2.1	4	0.40 ± 0.14				13.5 ± 0.6	2	0.31 ± 0.02
	ONE End_Ex	18.5 ± 5.7	7	0.47 ± 0.24	15.4	1	0.43	21.8 ± 13.7	4	0.56 ± 0.31
	TWO End_In	14.7	1	0.39						
	TWO End_Ex	21.6 ± 10.2	2	0.55 ± 0.26				22.2 ± 11.9	2	0.59 ± 0.37

ONE, one quarter of music tempo; TWO, two quarter of music tempo; End_In, end-inspiratory events; End_Ex, end-expiratory events; CON, control condition (no music); *n*, number of participants with significant entrainment (*p* < 0.05); blank spaces are provided when no significant entrainment was found for any of the participants.

**Table 4:** Number of observations of the entrainment analyses.

Form of Entrainment	Observed Parameter	Total	House	Latin	120 bpm	128 bpm	136 bpm
Music-locomotor	pedaling cycles	2376 ± 575	1155 ± 264	1221 ± 314	786 ± 194	789 ± 188	803 ± 197
Locomotor-breathing and Music-breathing	breaths	834 ± 196	416 ± 92	418 ± 107	268 ± 60	285 ± 78	282 ± 70

**Table 5:** Presence of music-breathing entrainment when considering one-quarter of music tempo as the music beat period.

Participants	Total	House	Latin	120 bpm	128 bpm	136 bpm
1	○	○	●			○●
2				○●		●
3	○	○	○●	○		
4	●		●			
5	○●		○●	○		
6	○	○	●	○		○
7				○●		
8	○		○	○		
9	○	●	○		○	○
10						○
11				●		
12		○		○●		

● significant (*p* < 0.05) music-breathing entrainment for end-inspiratory events, ○ significant (*p* < 0.05) music-breathing entrainment for end-expiratory events.

## Data Availability

The data presented in this study are available on request from the corresponding author.
